# PMS2 Expression With Combination of PD-L1 and TILs for Predicting Survival of Esophageal Squamous Cell Carcinoma

**DOI:** 10.3389/fonc.2022.897527

**Published:** 2022-07-05

**Authors:** Dongxian Jiang, Qi Song, Xiaojun Wei, Zixiang Yu, Yufeng Liu, Haixing Wang, Xingxing Wang, Jie Huang, Jieakesu Su, Yang Hong, Yifan Xu, Chen Xu, Yingyong Hou

**Affiliations:** ^1^ Department of Pathology, Zhongshan Hospital, Fudan University, Shanghai, China; ^2^ Shanghai Institute of Infectious Disease and Biosecurity, Fudan University, Shanghai, China

**Keywords:** DNA mismatch repair protein, PMS2, prognosis, PD-L1, TILs, esophageal squamous cell carcinoma (ESCC)

## Abstract

**Background:**

DNA mismatch repair (MMR) deficiency (dMMR) has been recognized as an important biomarker for immunotherapy in esophageal squamous cell carcinoma (ESCC), along with programmed death ligand 1 (PD-L1) expression and/or tumor-infiltrated lymphocytes (TILs). However, in ESCC, MMR protein assessment has not been well studied at present.

**Methods:**

A total of 484 ESCC tissues treated between 2007 and 2010, in our hospital, were enrolled. Immunohistochemical expression of MLH1, MSH2, MSH6, PMS2, and PD-L1 on tissue microarray specimens and clinicopathological features, including TILs, were analyzed retrospectively.

**Results:**

Out of the 484 studied cases, loss of MLH1, MSH2, MSH6, and PMS2 expression were found in 6.8%, 2.1%, 8.7%, and 4.8% patients, respectively. dMMR was found in 65 patients, 37 cases involved in one MMR protein, 17 cases involved in two proteins, 7 cases involved in three proteins, and 4 cases involved in four proteins. There was no significant survival difference between pMMR (MMR-proficient) and dMMR patients (*P*>0.05). However, 224 patients with low PMS2 expression had better DFS and OS than 260 patients with high PMS2 expression (*P*=0.006 for DFS and 0.008 for OS), which was identified as an independent prognostic factor in multivariate analyses. Positive PD-L1 expression was detected in 341 (70.5%) samples. In stage I-II disease, patients with PD-L1 expression had better DFS and OS than those without PD-L1 expression(*P*<0.05), which was not found in stage III-IV disease. With the ITWG system, 40.1% of cases were classified as high TILs. Patients in the high-TILs group tended to have better DFS (*P*=0.055) and OS (*P*=0.070) than those in the low-TILs group and the differences were statistically significant in pMMR, high MSH6, or PMS2 expression cases (*P*<0.05). Also, high PMS2 expression patients with both PD-L1 expression and high TILs, had similar DFS and OS compared with low PMS2 expression patients (*P*>0.05), which were much better than other high PMS2 expression patients.

**Conclusion:**

The expression level of MMR proteins could also be used as a prognostic factor in ESCC and PMS2 expression outperformed other MMR proteins for predicting survival. The combination of PD-L1 expression and TILs may lead to more efficient risk stratification of ESCC.

## Introduction

Esophageal cancer (EC) is the seventh most common cancer worldwide (1). According to the latest data in China, the age-standardized incidence rate by world standard population (ASIRW) of EC is 11.9/100,000, which is about 2 times the global level (2). In China, more than 90% EC is esophageal squamous cell carcinoma (ESCC), which contributes to 53% of the global cases. Therefore, China has carried the highest absolute burden of ESCC (3). Recently, immunotherapy with immune check point-blocking antibodies targeting programmed death 1 or programmed death ligand 1 (PD-1 or PD-L1) has improved the outcomes of EC patients, especially ESCC (4, 5). The interaction between PD-1 and its ligand (PD-L1) decreases the T-cell activity, resulting in tumor cell avoidance of the immune system. PD-L1 expression or tumor-infiltrated lymphocytes (TILs) can assist the tumor in escaping the immune system (6, 7). Multiple anti-PD1/PD-L1 drugs have been approved for use in solid tumors and PD-L1 expression and/or TILs have been approved as a companion diagnostic marker across different types of tumors, including ESCC.

DNA mismatch repair (MMR) deficiency (dMMR) has been recognized as a predictive biomarker for immunotherapy (8, 9). DNA dMMR is the third mechanism for the repair of a DNA lesion, which recognizes and repairs small loops within the duplex DNA that arise from nucleotide misincorporation, either by base-base mismatches or by insertion/deletion loops (10). The inactivation of MMR genes may present as the activation of oncogenes or the inactivation of tumor suppressor genes caused by microsatellite instability, or present as directly causing mutations in oncogenes, or tumor suppressor genes, thereby inducing carcinogenesis. The high tumor burden caused by dMMR can attract more TILs, increase the expression of PD-L1, and inhibit the immune response (11, 12). Although recent studies show the importance of dMMR in various tumors, limited research evaluating the status of MMR in ESCC has been conducted. Therefore, it is important to investigate the frequency of dMMR in ESCC.

To date, biochemical and genetic studies in eukaryotes have defined at least four genes (MLH1, MSH2, MSH6, and PMS2) whose protein products are required for DNA MMR (10, 13). dMMR can be identified by the lack of protein expression for any of the MMR genes detected by immunohistochemistry (IHC) (14). In the clinical practice, we found the level of MSH2, MSH6, PMS2, and MLH1 expression was heterogeneous within a tumor, varying from 0%–100%. In lung adenocarcinoma, high MSH2 expression was reported to be significantly correlated with increased tumor mutational burden, increased PD-L1 expression, and TILs (15). More and more researchers believe that examining MMR proteins, except for the purpose of MSI screening, might merit additional study as these proteins could provide information for predicting which patients were likely to benefit from immunotherapy (16–18). Given that the four proteins play critical roles in DNA MMR, we speculated high protein expression might also have some clinical significance in ESCC, which has not been well studied at present.

In this study, we aimed to determine IHC expression of the four MMR proteins in ESCC, to investigate the associations between MMR protein expression and clinicopathological parameters, including PD-L1 expression and TILs, and to explore their prognostic significance.

## Materials and Methods

### Patient Samples

A total of 484 patients who underwent resection for ESCC in our institution from 2007 to 2010 were included in this study. None of the patients had undergone pre-operative treatment for ESCC. Tissue microarrays (TMAs) were assembled from paraffin-embedded tissues using a manual tissue microarrayer (19). The clinical features of the cases and the macroscopic features of the tumors were obtained from the hospital archive system. Pathological profiles were re-evaluated by reviewing the hematoxylin/eosin (HE) slides. The clinicopathological features included age, sex, history of smoking, tumor size, tumor location, differentiation, vessel and nerve invasion, invasion depth, and lymph node metastasis. All patients were pathologically staged according to the 8th edition of TNM classification system of the American Joint Committee for Cancer. Follow-up information for the patients after surgery and treatment was provided by the referring clinicians or obtained directly from patients and their family members as standard procedure.

The study was conducted in accordance with the Declaration of Helsinki and with approval from the Ethics Committee of Zhongshan Hospital, Fudan University. Written informed consent was obtained from all the participants.

### IHC Analysis of MMR Expression

IHC for four MMR proteins (MLH1, MSH2, MSH6, and PMS2) and PD-L1 was performed on TMAs. IHC analysis of the above-mentioned proteins used the following primary antibodies: mouse anti-human MLH-1 (clone ES05; Dako, Glostrup, Denmark), mouse anti-human MSH-2 (clone FE11; Dako, Glostrup, Denmark), rabbit anti-human MSH-6 (clone EP49; Dako, Glostrup, Denmark), rabbit anti-human PMS2 (clone EP51; Dako, Glostrup, Denmark), and rabbit anti-human PD-L1 (SP142; OriGene Technologies, Maryland, USA), and was performed with the Ventana iView DAB Detection Kit on a BenchMark XT automated staining system (Ventana Medical Systems, Tucson, AZ).

### Assessment of Staining

The degree of expression by IHC was classified by three pathologists blinded to the data. Each MMR protein expression score in the nuclei of cancer cells was determined in 10% increments. Tumors showing a total absence of nuclear staining, with the adjacent normal tissue showing the presence of nuclear staining, were regarded as having lost MMR protein expression. Loss of one or more MMR (MLH-1, MSH-2, MSH-6, and PMS-2) protein expression was considered deficient (MMR-deficient, dMMR), otherwise it was considered normal (MMR-proficient, pMMR). PD-L1 expression is determined by the combined positive score (CPS). CPS is calculated by dividing the number of PD-L1 staining cells (tumor cells, lymphocytes, macrophages) by the total number of viable tumor cells and multiplying the fraction by 100. A lesion was considered PD-L1 positive if the CPS was ≥1.

### Tumor Infiltrating Lymphocytes Evaluation

With the standardized ITWG scoring methods (20), TIL amounts were determined using HE-stained tumor surgical sections. The density of TILs was assessed within the stromal compartment of the tumor mass and scored as a percentage of stromal area. Only TILs within the border of invasive tumors were assessed, so that dysplastic and *in situ* areas (including growth confined to the lamina propria) and inflammation outside the tumor borders were disregarded. TILs were judged to be present at a low level (TILs-low) if they comprised less than 10% of the stroma.

### Statistical Analysis

The interaction between MMR protein expression, PD-L1 expression, TILs, and clinicopathological characteristics were analyzed with the Chi-square test. Pearson correlation was used to evaluate the interaction and consistency of four MMR proteins. Disease-free survival (DFS) was estimated from the date of surgical resection to the date of the local recurrence, regional metastasis, distant metastasis, or death. Overall survival (OS) was measured from the date of operation to the time of death. Survival rates were calculated using the Kaplan-Meier method and the log-rank test was used to compare survival curves. Univariate and multivariate analyses were based on the Cox proportional hazards regression model. All variables with *P*<0.05 in the univariate analyses were entered into the multivariate analyses using a stepwise variable selection procedure to adjust for potential confounding factors. All statistical analyses were performed using SPSS 21.0, and *P*-values of less than 0.05 were considered statistically significant.

## Results

### Patient Characteristics

The clinicopathological features of the 484 ESCC patients are summarized in **Table 1**. The median age was 61.0 years (34-83 years). The cohort was comprised of 397 men and 87 women and the ratio of men to women was 4.6:1. A total of 189 patients were smokers and 292 were drinkers. The median Charlson index was 2 (range 0-7), with 31% of patients less than 2. The mean tumor size was 3.4cm. Of the ESCC tumor samples, 23 were located in the upper esophagus, 216 in the middle, and 223 in the lower area. Also, 19 tumors had good differentiation, 272 had moderate differentiation, and 193 had poor differentiation. Nerve infiltration was presented in 168 cases, vascular infiltration was presented in 109 cases, and lymph node metastases was recorded in 224 cases. According to the 8th AJCC TNM stage, 268 (55.4%) were diagnosed with Stage pI-II disease and 216 (44.6%) were diagnosed with Stage pIII-IVa disease. In our study, 60.1% patients had undergone the Sweet procedures, 20.2% the McKeown procedures, 15.3% the Ivor-Lewis procedures, and 4.3% minimally invasive procedures (thoracoscopy with esophagectomy and lymphadenectomy). Major complications were found in 88 (18.2%) patients and adjuvant therapy were performed in 96 (19.8%) patients. During the follow up, a total of 279 patients (54.4%) had disease progression and 277 patients (54.0%) died.

**Table 1:** Association between MMR expression and clinicopathological features of ESCC patients.

		MLH1			PMS2			MSH2			MSH6			MMR		
	No.	Low	High	*P*	Low	High	*P*	Low	High	*P*	Low	High	*P*	dMMR	pMMR	*P*
Age				0.896			0.903			0.674			0.747			0.653
<60	206	183	23		96	110		17	189		80	126		26	180	
>=60	278	248	30		128	150		26	252		112	166		39	239	
Sex				0.842			0.861			0.256			0.906			0.351
Female	87	78	9		41	46		5	82		35	52		9	78	
Male	397	353	44		183	214		38	359		157	240		56	341	
Smoking				0.835			0.002			0.557			0.571			0.706
No	295	262	33		120	175		28	267		120	175		41	254	
Yes	189	169	20		104	85		15	174		72	117		24	165	
Drinking				0.994			0.440			0.548			0.185			0.627
No	192	171	21		93	99		73	119		13	179		24	168	
Yes	292	260	32		131	161		119	173		30	262		41	251	
Tumor size				0.948			0.614			<0.001			0.780			0.057
<3.4cm	276	246	30		125	151		13	263		108	168		30	246	
>3.4cm	208	185	23		99	109		30	178		84	124		35	173	
Site				0.561			0.463			0.564			0.406			0.958
Upper	23	22	1		10	13		2	21		12	11		3	20	
Middle	216	191	25		109	107		16	200		82	134		28	188	
Low	223	197	26		100	123		23	200		90	133		31	192	
Differentiation				0.143			0.527			0.245			0.005			0.208
Well	19	19	0		11	8		0	19		12	7		0	19	
Moderate	272	245	27		127	145		28	244		118	154		39	233	
Poor	193	167	26		86	107		15	178		62	131		26	167	
Vessel invasion				0.744			0.438			0.376			0.471			0.283
No	375	333	42		170	205		31	344		152	223		47	328	
Yes	109	98	11		54	55		12	97		40	69		18	91	
Nerve invasion				0.624			0.667			0.326			0.945			0.201
No	316	283	33		144	172		31	285		125	191		47	269	
Yes	168	148	20		80	88		12	156		67	101		18	150	
Lymph node metastasis				0.668			0.051			0.211			0.201			0.772
No	260	233	27		131	129		27	233		110	150		36	224	
Yes	224	198	26		93	131		16	208		82	142		29	195	
pTNM Stage				0.693			0.068			0.305			0.381			0.998
I-II	268	240	28		134	134		27	241		111	157		36	232	
III-IV	216	191	25		90	126		16	200		81	135		29	187	
Disease progression				0.75			0.009			0.268			0.209			0.511
No	220	197	23		116	104		23	197		94	126		32	188	
Yes	264	234	30		108	156		20	244		98	166		33	231	
Death				0.928			0.009			0.293			0.196			0.559
No	222	198	24		117	105		23	199		95	127		32	190	
Yes	262	233	29		107	155		20	242		97	165		33	229	

### Expression of MMR Protein

**Figure 1:**
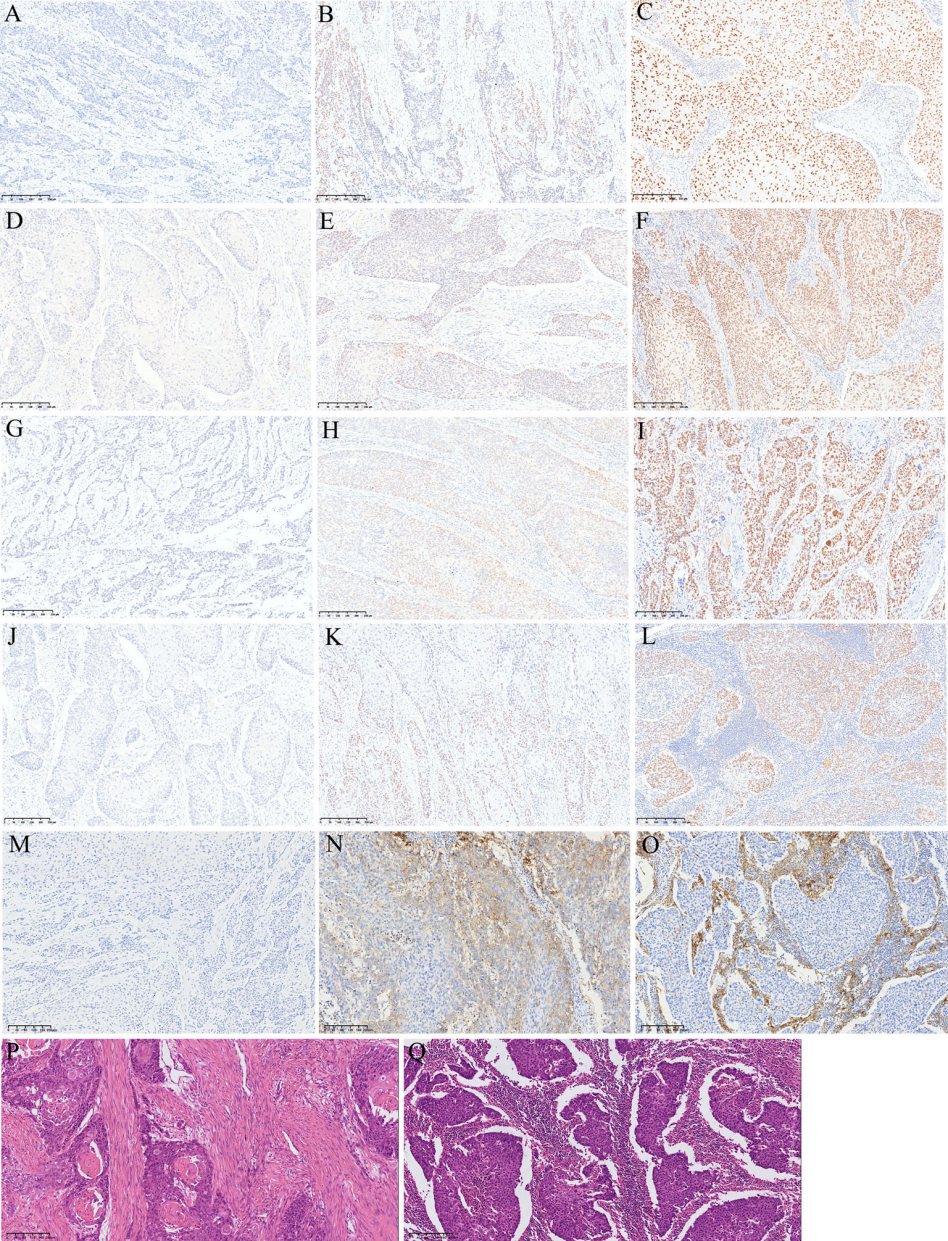
Representative images of HE and IHC. **(A)** Loss of MLH1 protein expression; **(B)** low MLH1 protein expression; **(C)** high MLH1 protein expression; **(D)** loss of PMS2 protein expression; **(E)** low PMS2 protein expression; **(F)** high PMS2 protein expression; **(G)** loss of MSH2 protein expression; **(H)** low MSH2 protein expression; **(I)** high MSH2 protein expression; **(J)** loss of MSH6 protein expression; **(K)** low MSH6 protein expression; **(L)** high MSH6 protein expression; **(M)** negative PD-L1 expression; **(N)** positive PD-L1 expression in tumor cells; **(O)** positive PD-L1 expression in tumor associated immune cells; **(P)** low TILs; **(Q)** high TILs.

IHC staining results of four MMR proteins (MLH1, MSH2, MSH6, and PMS2) are shown in **Figures 1A-L**. The levels of MMR protein expression among patient tumor specimens were highly variable. The median percentage for MLH1 was 15 (interquartile range [IRQ], 10-30), 60 for MSH2 (IQR, 40-70), 20 for MSH6 (IQR, 10-40), and 30 for PMS2 (IQR, 15-50). The optimal cutoff value for disease progression was 45, 55, 1.5, and 22.5 for MLH1, MSH2, MSH6, and PMS2, respectively, according to the ROC curve analysis. Low expression of MLH1, MSH2, MSH6, and PMS2 were found in 89%, 39.7%, 8.9% and 46.3% patients, respectively. A significant correlation among four MMR proteins was observed (*P*<0.001) and a better consistency was found between MLH1 and PMS2 (Pearson correlation=0.626), MSH2, and MSH6 (Pearson correlation=0.623) (**Table 2**).

**Table 2:** Correlation analysis of the four MMR protein expressions.

	IHC score	
	*P*	Pearson correlation
MLH1 VS. MSH2	<0.001	0.453
MLH1 VS. MSH6	<0.001	0.519
MLH1 VS. PMS2	<0.001	0.626
MSH2 VS. MSH6	<0.001	0.623
MSH2 VS. PMS2	<0.001	0.467
MSH6 VS. PMS2	<0.001	0.455

0.6<Pearson correlation<0.8, a moderate correlation existed.

Loss of MLH1, MSH2, MSH6, and PMS2 expression were found in 33 (6.8%), 10 (2.1%), 42 (8.7%), and 23 (4.8%) patients, respectively. dMMR was found in 65 patients (13.4%), among whom 4 were co-deficient in MLH1, MSH2, MSH6, and PMS2; 4 patients were co-deficient in MLH1, MSH2, and MSH6; 3 patients were co-deficient in MLH1, MSH6, and PMS;, 8 patients were co-deficient in MLH1 and PMS2; 5 patients were co-deficient in MLH1 and MSH6; 2 patients were co-deficient in MSH2 and MSH6; 2 patients were co-deficient in MSH6 and PMS2; 22 patients were deficient in MSH6; 9 patients were deficient in MLH1; and 6 patients were deficient in PMS2.

### Association of MMR Status With Clinicopathological Characteristics

The relationship between clinicopathologic features and MMR status is listed in **Table 1**. High MSH2 expression was associated with tumor size and low expression occurred more frequently in tumors with larger size (*P<*0.001), which was not found in MLH1, MSH2, and PMS2. High PMS2 expression was associated with smoking, disease progression, and death. Low expression occurred more frequently in the smoking group (*P*=0.002) and patients without disease progression or death (*P*=0.009), which was not found in MLH1, MSH2, and MSH6.

**Figure 2:**
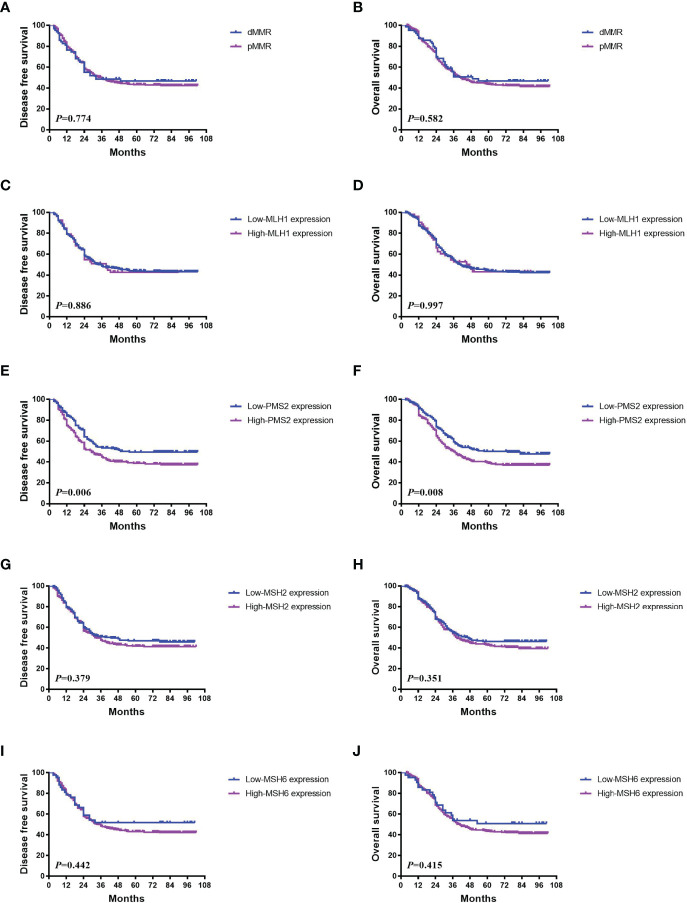
Association between MMR status and survival in ESCC. **(A, B)** There was no significant survival difference between pMMR and dMMR patients (*P*>0.05). **(C, D)** No differences in DFS (*P* = 0.886) and OS (*P* = 0.997) were found between patients with low MLH1 expression and high expression. **(E, F)** 224 patients with low PMS2 expression had better DFS and OS than 260 patients with high PMS2 expression (*P* = 0.006 for DFS and 0.008 for OS). **(G–J)** Similar tendencies were also observed in those with low MSH2 expression (*P* = 0.379 for DFS and 0.351 for OS), and low MSH6 expression (*P* = 0.442 for DFS and 0.415 for OS).

There was no significant survival difference between pMMR and dMMR patients (*P*>0.05) (**Figures 2A, B**). However, 224 patients with low PMS2 expression had better DFS and OS than 260 patients with high PMS2 expression (*P*=0.006 for DFS and 0.008 for OS, **Figures 2E, F**). A similar tendency was also observed in those with low MSH6 expression (*P*=0.442 for DFS and 0.415 for OS, **Figures 2I, J**). No differences in survival were found between patients with low MLH1 expression and high MLH1 expression (*P*=0.886 for DFS and 0.997 for OS) (**Figures 2C, D**) and between patients with low MSH2 expression and high MSH2 expression (*P*=0.379 for DFS and 0.351 for OS, **Figures 2G, H**). There was no association between PMS2 deficiency and DFS (*P*=0.964) or OS (*P*=0.906) (**Supplementary Figures 1A, B**).

### Expression of PD-L1 and Correlation With MMR Status

Positive PD-L1 expression was detected in 341 (70.5%) samples (**Figures 1M, O**). The relationship between clinicopathological features and PD-L1 expression is listed in **Table 3**. PD-L1 expression was significantly associated with high MSH2 expression, high MSH6 expression, and high PMS2 expression. PD-L1 expression also tended to be associated with high MLH1 expression and pMMR. No significant correlations were found between PD-L1 expression and patient age (*P*=0.819), sex (*P*=0.082), smoking (*P*=0.396), drinking (*P*=0.171), tumor size (*P*=0.927), tumor site (*P*=0.682), differentiation (*P*=0.941), vessel and nerve invasion (*P*=0.365 and 0.071), lymph node metastasis (*P*=0.403), and pTNM stages (*P*=0.716).

**Table 3:** Association between PD-L1 expression, TILs, and clinicopathological features of ESCC patients.

	PD-L1 (CPS≧1)		TILs (≧10)			
	Negative	Positive	*P*	Low	High	*P*
Age			0.819			0.915
<60	62	144		124	82	
>=60	81	197		166	112	
Sex			0.082			0.027
Female	19	68		43	44	
Male	124	273		247	150	
Smoking			0.396			0.600
No	83	212		174	121	
Yes	60	129		116	73	
Drinking			0.171			0.339
No	50	142		110	82	
Yes	93	199		180	112	
Tumor size			0.927			0.007
<3.4cm	82	194		151	125	
>3.4cm	61	147		139	69	
Site			0.682			0.451
Upper	5	18		14	9	
Middle	64	152		121	95	
Low	68	155		138	85	
Differentiation			0.941			0.460
Well	5	14		9	10	
Moderate	80	192		167	105	
Poor	58	135		114	79	
Vessel invasion			0.365			0.550
No	107	268		222	153	
Yes	36	73		68	41	
Nerve invasion			0.071			0.398
No	102	214		185	131	
Yes	41	127		105	63	
Lymph node metastasis			0.403			0.481
No	81	179		152	108	
Yes	62	162		138	86	
pTNM Stage			0.716			0.220
I-II	81	187		154	114	
III-IV	62	154		136	80	
MLH1 expression			0.071			0.021
Low	133	298		266	165	
High	10	43		24	29	
MSH2 expression			0.036			0.453
Low	67	125		119	73	
High	76	216		171	121	
MSH6 expression			0.001			0.003
Low	22	21		35	8	
High	121	320		255	186	
PMS2 expression			0.01			0.207
Low	79	145		141	83	
High	64	196		149	111	
MMR			0.09			0.003
dMMR	25	40		50	15	
pMMR	118	301		240	179	
PD-L1 (CPS≧1)						<0.001
Negative				111	32	
Positive				179	162	

**Figure 3:**
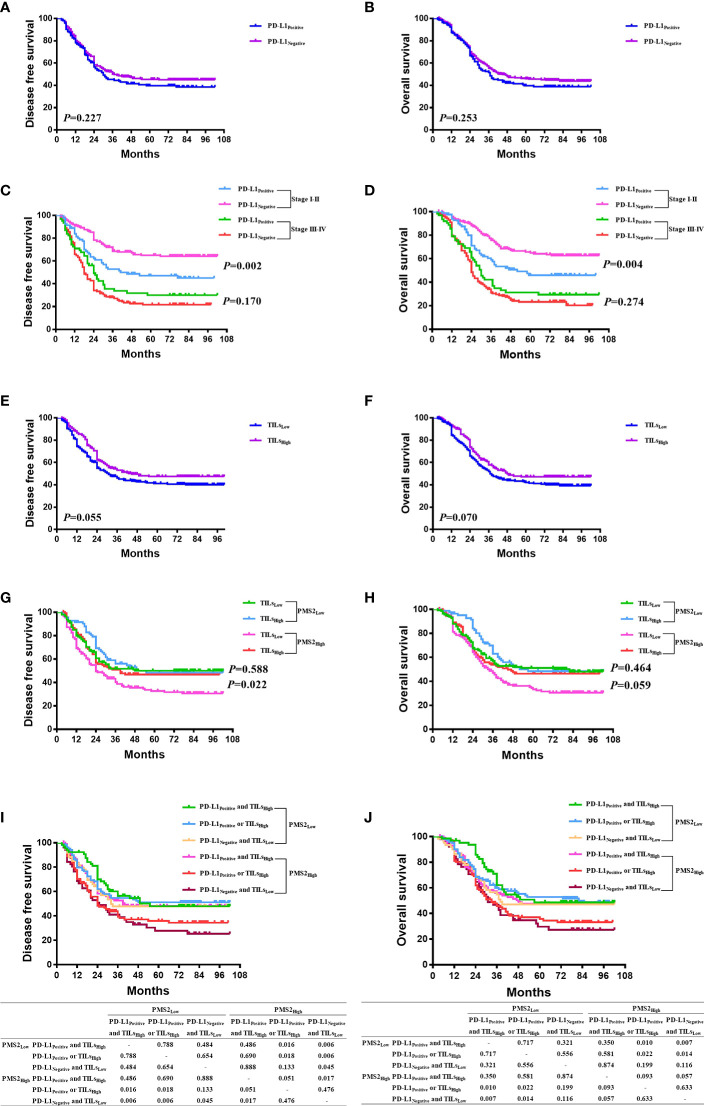
The prognostic significance of MMR expression, PD-L1 expression, and TILs. **(A, B)** There was no association between PD-L1 expression and DFS or OS (*P* > 0.05); **(C, D)** In stage I-II of disease, patients with PD-L1 expression had better DFS and OS than those without PD-L1 expression (*P* < 0.05), which were not found in stage III-IV of disease (*P* > 0.05); **(E, F)** Patients in the high-TILs group tended to have better DFS (*P*=0.055) and OS (*P* = 0.070) than those in the low-TILs group; **(G, H)** In high PMS2 expression, patients in the high-TILs group have better DFS (*P* = 0.022) and OS (*P* = 0.059) than those in the low-TILs group, which were not found in the low PMS2 expression group; **(I, J)** In 260 patients with high PMS2 expression, the order from better prognosis to poorer survival is 98 patients with both PD-L1 expression and high TILs, 111 patients with either PD-L1 expression or high TILs, and 51 patients with neither PD-L1 expression nor high TILs. However, in 224 patients with low PMS2 expression, there was no survival difference among the three cohorts (*P* > 0.05).

There was no association between PD-L1 expression and DFS or OS (*P*>0.05) in the Kaplan-Meier analysis (**Figures 3A, B**). Survival analysis were also conducted in I-II stage and III-IV stage disease, separately. In stage I-II disease, patients with PD-L1 expression had better DFS and OS than those without PD-L1 expression (*P*<0.05), which were not found in stage III-IV disease (*P>*0.05, **Figures 3C, D**). In subgroup analyses for patients with high PMS2 expression, patients with PD-L1 expression tended to have better DFS (*P*=0.103) and OS (*P*=0.190) than those without PD-L1 expression, which was not found in the subgroup analyses for patients with low PMS2 expression (**Supplementary Figures 2A, B**).

### Tumor Infiltrating Lymphocytes and MMR Status

TILs were scored on 484 patients. With the ITWG system, 290 cases (59.9%) were classified as low TILs and 194 (40.1%) as high TILs (**Figures 1P, Q**). High TILs scores were significantly associated with women (*P*=0.027), smaller tumor size (*P*=0.007), high MLH1 expression (*P*=0.021), high MSH6 expression (*P*=0.003), pMMR (*P*=0.003), and PD-L1 expression (*P*<0.05). No significant differences in TILs scores were observed for age (*P*=0.915), smoking (*P*=0.600), drinking (*P*=0.339), tumor site (*P*=0.451), differentiation (*P*=0.460), vessel invasion (*P*=0.550), nerve invasion (*P*=0.398), lymph node metastasis (*P*=0.481), and pTNM stage (*P*=0.220) (**Table 3**).

Patients in the high-TIL group tended to have better DFS (*P*=0.055) and OS (*P*=0.070) than those in the low-TIL group (**Figures 3E, F**). This survival benefit was statistically significant in the subgroup analyses for pMMR cases (*P*=0.031 for DFS, *P*=0.043 for OS), but not for dMMR cases (*P*=0.860 for DFS, *P*=0.952 for OS); in subgroup analyses for high MSH6 expression (*P*=0.015 for DFS, *P*=0.021 for OS), but not for low MSH6 expression (*P*=0.203 for DFS, *P*=0.243 for OS); in subgroup analyses for high PMS2 expression (*P*=0.022 for DFS), but not for low PMS2 expression (*P*=0.588 for DFS, **Figures 3G, H**).

### PMS2 Expression Outperformed Other MMR Expression for Predicting Survival

In univariate analyses, differentiation, vessel invasion, nerve invasion, pTNM stage, and PMS2 expression were significantly associated with DFS. Vessel invasion, nerve invasion, pTNM stage, and PMS2 expression were also significantly associated with OS. Multivariate analyses showed significant association between decreased survival and high PMS2 expression (hazard ratio [HR]=1.315, 95% confidence interval [CI]: 1.026-1.684, *P*=0.030 for DFS; HR=1.339, 95% CI: 1.044-1.717, *P*=0.021 for OS), along with TNM stage (HR=2.560, 95% CI: 1.962-3.339, *P*<0.001 for DFS; HR=2.609, 95% CI: 2.001-3.404, *P*<0.001 for OS) (**Table 4**).

**Table 4:** Univariate and multivariate analyses of prognostic factors for survival.

	DFS		OS	
	*P*	HR (95% CI)	*P*	HR (95% CI)
**Univariate factor analysis**				
Sex	0.296	1.183 (0.863-1.622)	0.291	1.187 (0.863-1.632)
Age	0.817	1.029 (0.806-1.314)	0.596	1.069 (0.836-1.365)
Smoking	0.254	1.154 (0.902-1.475)	0.192	1.178 (0.921-1.506)
Drinking	0.104	1.231 (0.958-1.581)	0.131	1.214 (0.944-1.561)
Charlson index	0.198	1.191 (0.913-1.555)	0.133	1.228 (0.939-1.606)
Tumor Size	0.167	1.187 (0.931-1.514)	0.077	1.245 (0.976-1.589)
Tumor Location	0.646	0.953 (0.774-1.172)	0.831	0.977 (0.793-1.205)
Differentiation	0.030	1.274 (1.023-1.587)	0.081	1.217 (0.976-1.516)
Vessel invasion	0.001	1.568 (1.203-2.043)	0.002	1.526 (1.167-1.996)
Nerve invasion	0.021	1.340 (1.046-1.716)	0.004	1.445 (1.128-1.851)
Invasive Depth	<0.001	1.634 (1.312-2.035)	<0.001	1.769 (1.406-2.225)
Lymph node metastasis	<0.001	2.717 (2.116-3.489)	<0.001	2.752 (2.141-3.538)
pTNM stage	<0.001	2.766 (2.157-3.546)	<0.001	2.790 (2.174-3.580)
Operative approach	0.692	0.977 (0.873-1.094)	0.867	0.990 (0.884-1.109)
Complication	0.176	0.793 (0.566-1.110)	0.090	0.742 (0.526-1.047)
Adjuvant therapy	0.093	1.279 (0.959-1.706)	0.258	1.185 (0.883-1.589)
MLH1 expression	0.888	1.028 (0.703-1.503)	0.997	0.999 (0.679-1.470)
MSH2 expression	0.388	1.116 (0.870-1.433)	0.358	1.125 (0.875-1.446)
MSH6 expression	0.451	1.192 (0.755-1.880)	0.422	1.206 (0.764-1.902)
PMS2 expression	0.007	1.398 (1.094-1.787)	0.009	1.391 (1.087-1.780)
dMMR	0.778	1.054 (0.732-1.518)	0.587	1.106 (0.768-1.594)
PD-L1expression	0.235	0.855 (0.660-1.108)	0.259	0.861 (0.664-1.117)
TILs	0.060	0.786 (0.612-1.010)	0.074	0.795 (0.618-1.023)
**Mutivariate factor analysis**				
Differentiation	0.360	1.112 (0.886-1.394)	-	-
Vessel invasion	0.603	1.078 (0.812-1.432)	0.747	1.048 (0.788-1.393)
Nerve invasion	0.365	1.125 (0.872-1.453)	0.112	1.230 (0.953-1.589)
pTNM stage	<0.001	2.560 (1.962-3.339)	<0.001	2.609 (2.001-3.404)
PMS2 expression	0.030	1.315 (1.026-1.684)	0.021	1.339 (1.044-1.717)

Survival analysis was conducted in patients with high PMS2 expression and low PMS2 expression, with combination of PD-L1 expression and TILs (**Figures 3I, J**). In 260 patients with high PMS2 expression, the order from better prognosis to poorer survival is 98 patients with both PD-L1 expression and high TILs, 111 patients with either PD-L1 expression or high TILs, and 51 patients with neither PD-L1 expression nor high TILs. While in 224 patients with low PMS2 expression, there was no survival difference among the three cohorts (*P*>0.05). In addition, high PMS2 expression patients with both PD-L1 expression and high TILs had similar DFS and OS with low PMS2 expression patients (*P*>0.05).

## Discussion

MMR proteins play important role in maintaining the structure and function of DNA. The error rate during replication increased one hundredfold to one thousandfold with the loss of this repair mechanism (13, 21). dMMR is frequently observed in digestive cancers, including colorectal cancers, gastric cancers, and esophageal adenocarcinoma (10, 22). However, MMR expression has not been well analyzed in ESCC. Recently, another role of the MMR system has been revealed to be associated with immunotherapy in tumors of different types (9, 23). Therefore, knowledge about MMR features in ESCC may provide important information about how ESCC should be managed in the future. To the best of our knowledge, our study is the first to systematically analyze the expression of four MMR proteins in a large cohort of ESCC patients without neoadjuvant therapy.

In our cohort, 13.4% of tumors showed loss of one or more MMR protein (MLH1, MSH2, MSH6, and PMS2) expressions, which was consistent with the results in previous studies (24, 25). Among the 65 tumors that showed a loss of MMR protein expressions, 56.9% (37/65) were involved in one MMR protein, 26.2% (17/65) were involved in two proteins, 10.8% (7/65) were involved in three proteins, and 6.1% (4/65) were involved in four proteins. The loss of MSH6 expression (8.7%) was more frequent than MLH1 (6.8%), MSH2 (2.1%), and PMS2 (4.8%). There was a strong correlation between MSH6 and MSH2, and MLH1 and PMS2, which is consistent with the fact that MLH1 protein dimerizes with the PMS2 protein and the MSH2 protein binds to the MSH6 protein, which play their roles in the MMR process as complex (13). Several studies also demonstrated the difference of mostly affected MMR genes and the combination pattern of defects in other type of tumors. Annukka et al. found the most commonly affected genes were MLH1 in endometrial carcinoma (26). Zekri et al. observed the most frequently affected genes were MSH2, MSH6, and MLH1 in hepatocellular carcinoma (27). Therefore, it seems possible that different patterns of MMR protein abnormalities might be found in different tumor types.

The present study demonstrates there was no difference in the prognosis between dMMR and pMMR tumors. However, in few studies with small-size samples, dMMR was reported to be associated with poor prognosis (28). We further compared the levels of four MMR protein expressions in our ESCC, and found high PMS2 expression was independently a prognostic factor with multivariate survival analyses. Namely, 224 patients with low PMS2 expression had better DFS and OS than 260 patients with high PMS2 expression. Some studies also revealed that PMS2 expression might be an important prognostic factor. In oral squamous cell carcinoma, Decker et al. found high PMS2 expression significantly increased the risk of death for patients aged 60 years or younger (29). Alixanna et al. recognized PMS2 elevation as a prognostic marker in pre-neoplastic and prostate cancer lesions (30). It is reported that overexpression of PMS2 can disrupt the cytotoxic signaling pathway and lead to non-productive interactions with pro-apoptotic factors, thus enhancing tolerance to DNA damage (31).

dMMR tumors were found to present more frequent PD-L1 positivity in some research (11, 32). However, it is not known whether these findings are universal across various subgroups of dMMR carcinomas. There was no correlation between MMR status and PD-L1expression in ovarian cancer (33). In breast cancer, a substantial proportion of patients without PD-L1 expression showed complete/partial loss of MMR (34). In our cohort, PD-L1 expression was associated with high MMR expression or pMMR. As to the prognostic significance of PD-L1 expression, the finding was conflicting in different studies. In gastric cancer, higher PD-L1 level (CPS≥1) had a significantly better PFS (progression free survival) and OS (35). In sinonasal squamous cell carcinoma, PD-L1 expression was significantly associated with worse OS (36). In our study, patients with PD-L1 expression had better DFS and OS than those without PD-L1 expression in stage I-II disease but not in stage III-IV disease, which was consistent with a previous study of ESCC (37).

As immunologically hot tumors, dMMR tumors are thought to be heavily infiltrated by TILs. However, it is surprising that more and more studies found there was no statistically significant association between TILs and dMMR. In breast cancer, the authors revealed that MSI-H cancers do not correspond to TIL-high tumors (38). In endometrial cancer, Dong et al. found pMMR tumors harbored increased density of TILs (39). In ESCC, we identified high TILs were associated with pMMR, high MLH1 expression, and high MSH6 expression. TILs also showed a significant correlation with PD-L1 expression, as reported in other tumors (40). Patients in the high-TIL group tended to have better survival than those in the low-TIL group and this survival benefit was statistically significant in the subgroup analyses for pMMR cases and high MSH6 or PMS2 expression cases. We speculated some difference might be exited between different tumor types or cohorts, for example one research study found the composition and prognosis of TILs between Caucasian and Asian lung cancer patients was quite different (41). The prognostic significance of high TILs and PD-L1 expression were also analyzed according to PMS2 status. In patients with high PMS2 expression (poorer survival), those with both high TILs and PD-L1 expression had better outcomes than those with either high TILs or PD-L1 expression and those with neither high TILs nor PD-L1 expression, which were not found in patients with lower PMS2 expression (better survival). Moreover, high PMS2 expression patients with both PD-L1 expression and high TILs, had similar prognosis with low PMS2 expression patients, which demonstrated high PMS2 expression, with combination of PD-L1 expression and high TILs, could more accurately identify high-risk groups. Some results were consistent with the finding in non-small cell lung cancer (42). Therefore, it is also important to evaluate TILs and PD-L1 status in pMMR ESCC for accurate risk classification.

In conclusion, the present study adds valuable information to the current literature because it investigates the expression pattern of four MMR proteins in a larger cohort of ESCC patients. There was no significant survival difference between pMMR and dMMR patients. However, high PMS2 expression was significantly correlated with poorer outcomes and was verified as an independent prognostic factor. The combination of PD-L1 expression and TILs could enable us to differentiate patients’ survival outcomes in more detail. High PMS2 expression patients with both PD-L1 expression and high TILs had similar prognosis with low PMS2 expression patients, which were much better than high PMS2 expression patients. The results of the present study illustrate that the expression level of MMR proteins could also be used as prognostic factor in ESCC. Also, TILs and PD-L1 status might lead to more efficient risk stratification of ESCC.

## Data Availability Statement

All data generated or analysed during this study are included in this published article. The names of the repository/repositories and accession number(s) can be found in the article/**Supplementary Material**.

## Ethics Statement

The studies involving human participants were reviewed and approved by Ethics Committee of Zhongshan Hospital, Fudan University. The patients/participants provided their written informed consent to participate in this study. Written informed consent was obtained from all the participants.

## Author Contributions

YH and CX performed study concept and design; DJ, QS, XWe and ZY performed development of methodology and writing; YH and CX review and revision of the paper; DJ, QS, YL, HW, XWa, JH, and YH provided acquisition, analysis and interpretation of data, and statistical analysis; JS and YX provided technical and material support. All authors read and approved the final paper.

## Funding

This work was financially supported by Shanghai Municipal Health Commission (No. 20214Y0275), National Natural Science Foundation of China (No. 81702372), Shanghai Municipal Commission of Science and Technology (No. 19441904000), Shanghai Municipal Key Clinical Specialty (No. shslczdzk01302), and Shanghai Science and Technology Development Fund (No. 19MC1911000).

## Conflict of Interest

The authors declare that the research was conducted in the absence of any commercial or financial relationships that could be construed as a potential conflict of interest.

## Publisher’s Note

All claims expressed in this article are solely those of the authors and do not necessarily represent those of their affiliated organizations, or those of the publisher, the editors and the reviewers. Any product that may be evaluated in this article, or claim that may be made by its manufacturer, is not guaranteed or endorsed by the publisher.

## Supplementary Material

Supplementary Figure 1
**(A, B)** There was no association between PMS2 deficiency and DFS (*P*=0.964) or OS (*P*=0.906).

Supplementary Figure 2
**(A, B)** In subgroup analyses for patients with high PMS2 expression, patients with PD-L1 expression tended to have better DFS (*P*=0.103) and OS (*P*=0.190) than those without PD-L1 expression, which were not found in subgroup analyses for patients with low PMS2 expression.

## References

[1] Sung H Ferlay J Siegel RL Laversanne M Soerjomataram I Jemal A. Global Cancer Statistics 2020: GLOBOCAN Estimates of Incidence and Mortality Worldwide for 36 Cancers in 185 Countries. CA: Cancer J Clin (2021) 71(3):209–49. doi: 10.3322/caac.21660 33538338

[2] The Global, Regional, and National Burden of Oesophageal Cancer and its Attributable Risk Factors in 195 Countries and Territories, 1990-2017: A Systematic Analysis for the Global Burden of Disease Study 2017. Lancet Gastroenterol Hepatol (2020) 5(6):582–97. doi: 10.1016/S2468-1253(20)30007-8 PMC7232026 32246941

[3] Abnet CC Arnold M Wei WQ. Epidemiology of Esophageal Squamous Cell Carcinoma. Gastroenterology (2018) 154(2):360–73. doi: 10.1053/j.gastro.2017.08.023 PMC5836473 28823862

[4] Kojima T Shah MA Muro K Francois E Adenis A Hsu CH. Randomized Phase III KEYNOTE-181 Study of Pembrolizumab Versus Chemotherapy in Advanced Esophageal Cancer. J Clin Oncol (2020) 38(35):4138–48. doi: 10.1200/JCO.20.01888 33026938

[5] Kato K Cho BC Takahashi M Okada M Lin CY Chin K. Nivolumab Versus Chemotherapy in Patients With Advanced Oesophageal Squamous Cell Carcinoma Refractory or Intolerant to Previous Chemotherapy (ATTRACTION-3): A Multicentre, Randomised, Open-Label, Phase 3 Trial. Lancet Oncol (2019) 20(11):1506–17. doi: 10.1016/S1470-2045(19)30626-6 31582355

[6] Pérez-Ruiz E Melero I Kopecka J Sarmento-Ribeiro AB García-Aranda M De Las Rivas J. Cancer Immunotherapy Resistance Based on Immune Checkpoints Inhibitors: Targets, Biomarkers, and Remedies. Drug Resist Updates (2020) 53:100718. doi: 10.1016/j.drup.2020.100718 32736034

[7] Kockx MM McCleland M Koeppen H. Microenvironmental Regulation of Tumour Immunity and Response to Immunotherapy. J Pathol (2021) 254(4):374–83. doi: 10.1002/path.5681 PMC8252752 33846997

[8] Le DT Uram JN Wang H Bartlett BR Kemberling H Eyring AD. PD-1 Blockade in Tumors With Mismatch-Repair Deficiency. New Engl J Med (2015) 372(26):2509–20. doi: 10.1056/NEJMoa1500596 PMC4481136 26028255

[9] Le DT Durham JN Smith KN Wang H Bartlett BR Aulakh LK. Mismatch Repair Deficiency Predicts Response of Solid Tumors to PD-1 Blockade. Sci (New York NY) (2017) 357(6349):409–13. doi: 10.1126/science.aan6733 PMC5576142 28596308

[10] Basso D Navaglia F Fogar P Zambon CF Greco E Schiavon S. DNA Repair Pathways and Mitochondrial DNA Mutations in Gastrointestinal Carcinogenesis. Clinica chimica acta; Int J Clin Chem (2007) 381(1):50–5. doi: 10.1016/j.cca.2007.02.020 17397816

[11] Svensson MC Borg D Zhang C Hedner C Nodin B Uhlén M. Expression of PD-L1 and PD-1 in Chemoradiotherapy-Naïve Esophageal and Gastric Adenocarcinoma: Relationship With Mismatch Repair Status and Survival. Front Oncol (2019) 9:136. doi: 10.3389/fonc.2019.00136 30931254 PMC6425870

[12] Bai Z Zhou Y Ye Z Xiong J Lan H Wang F. Tumor-Infiltrating Lymphocytes in Colorectal Cancer: The Fundamental Indication and Application on Immunotherapy. Front Immunol (2021) 12:808964. doi: 10.3389/fimmu.2021.808964 35095898 PMC8795622

[13] Sameer AS Nissar S Fatima K. Mismatch Repair Pathway: Molecules, Functions, and Role in Colorectal Carcinogenesis. Eur J Cancer Prev (2014) 23(4):246–57. doi: 10.1097/CEJ.0000000000000019 24614649

[14] McConechy MK Talhouk A Li-Chang HH Leung S Huntsman DG Gilks CB. Detection of DNA Mismatch Repair (MMR) Deficiencies by Immunohistochemistry can Effectively Diagnose the Microsatellite Instability (MSI) Phenotype in Endometrial Carcinomas. Gynecol Oncol (2015) 137(2):306–10. doi: 10.1016/j.ygyno.2015.01.541 25636458

[15] Jia M Yao L Yang Q Chi T. Association of MSH2 Expression With Tumor Mutational Burden and the Immune Microenvironment in Lung Adenocarcinoma. Front Oncol (2020) 10:168. doi: 10.3389/fonc.2020.00168 32154170 PMC7046689

[16] Caccese M Ius T Simonelli M Fassan M Cesselli D Dipasquale A. Mismatch-Repair Protein Expression in High-Grade Gliomas: A Large Retrospective Multicenter Study. Int J Mol Sci (2020) 21(18):6716. doi: 10.3390/ijms21186716 PMC7555820 32937743

[17] Maiuri AR Peng M Podicheti R Sriramkumar S Kamplain CM Rusch DB. Mismatch Repair Proteins Initiate Epigenetic Alterations During Inflammation-Driven Tumorigenesis. Cancer Res (2017) 77(13):3467–78. doi: 10.1158/0008-5472.CAN-17-0056 PMC5516887 28522752

[18] Gambichler T Abu Rached N Tannapfel A Becker JC Vogt M Skrygan M. Expression of Mismatch Repair Proteins in Merkel Cell Carcinoma. Cancers (2021) 13(11):2524. doi: 10.3390/cancers13112524 34063983 PMC8196722

[19] Shi Y He D Hou Y Hu Q Xu C Liu Y. An Alternative High Output Tissue Microarray Technique. Diagn Pathol (2013) 8:9. doi: 10.1186/1746-1596-8-9 23336116 PMC3599403

[20] Salgado R Denkert C Demaria S Sirtaine N Klauschen F Pruneri G. The Evaluation of Tumor-Infiltrating Lymphocytes (TILs) in Breast Cancer: Recommendations by an International TILs Working Group 2014. Ann Oncol (2015) 26(2):259–71. doi: 10.1093/annonc/mdu450 PMC6267863 25214542

[21] Jun SH Kim TG Ban C. DNA Mismatch Repair System. Classical Fresh Roles FEBS J (2006) 273(8):1609–19. doi: 10.1111/j.1742-4658.2006.05190.x 16623698

[22] Svrcek M Lascols O Cohen R Collura A Jonchere V Flejou JF. MSI/MMR-Deficient Tumor Diagnosis: Which Standard for Screening and for Diagnosis? Diagnostic Modalities for the Colon and Other Sites: Differences Between Tumors. Bull du Cancer (2019) 106(2):119–28. doi: 10.1016/j.bulcan.2018.12.008 30713006

[23] Diaz LA Jr. Le DT. PD-1 Blockade in Tumors With Mismatch-Repair Deficiency. New Engl J Med (2015) 373(20):1979. doi: 10.1056/NEJMc1510353 26559582

[24] Kishi K Doki Y Yano M Yasuda T Fujiwara Y Takiguchi S. Reduced MLH1 Expression After Chemotherapy is an Indicator for Poor Prognosis in Esophageal Cancers. Clin Cancer Res (2003) 9(12):4368–75. 14555508

[25] Lee HK Kwon MJ Ra YJ Lee HS Kim HS Nam ES. Significance of Druggable Targets (PD-L1, KRAS, BRAF, PIK3CA, MSI, and HPV) on Curatively Resected Esophageal Squamous Cell Carcinoma. Diagn Pathol (2020) 15(1):126. doi: 10.1186/s13000-020-01045-4 33054840 PMC7557072

[26] Pasanen A Loukovaara M Bützow R. Clinicopathological Significance of Deficient DNA Mismatch Repair and MLH1 Promoter Methylation in Endometrioid Endometrial Carcinoma. Modern Pathol (2020) 33(7):1443–52. doi: 10.1038/s41379-020-0501-8 32060377

[27] Zekri AR Sabry GM Bahnassy AA Shalaby KA Abdel-Wahabh SA Zakaria S. Mismatch Repair Genes (Hmlh1, Hpms1, Hpms2, GTBP/hMSH6, Hmsh2) in the Pathogenesis of Hepatocellular Carcinoma. World J Gastroenterol (2005) 11(20):3020–6. doi: 10.3748/wjg.v11.i20.3020 PMC4305833 15918183

[28] Uehara H Miyamoto M Kato K Cho Y Kurokawa T Murakami S. Deficiency of Hmlh1 and Hmsh2 Expression is a Poor Prognostic Factor in Esophageal Squamous Cell Carcinoma. J Surg Oncol (2005) 92(2):109–15. doi: 10.1002/jso.20332 16231369

[29] Decker JM Filho OV Freitas MO Silva-Fernandes IJ Dantas TS Campêlo CS. PMS2: A Potential Prognostic Protein Marker in Oral Squamous Cell Carcinoma. Medicina Oral Patologia Oral y Cirugia Bucal (2021) 26(4):e451–e8. doi: 10.4317/medoral.24303 PMC8254887 33247565

[30] Norris AM Woodruff RD D'Agostino RB Jr. Clodfelter JE Scarpinato KD. Elevated Levels of the Mismatch Repair Protein PMS2 are Associated With Prostate Cancer. Prostate (2007) 67(2):214–25. doi: 10.1002/pros.20522 17044039

[31] Gibson SL Narayanan L Hegan DC Buermeyer AB Liskay RM Glazer PM. Overexpression of the DNA Mismatch Repair Factor, PMS2, Confers Hypermutability and DNA Damage Tolerance. Cancer Lett (2006) 244(2):195–202. doi: 10.1016/j.canlet.2005.12.009 16426742

[32] Zong L Sun Z Mo S Lu Z Yu S Xiang Y. PD-L1 Expression in Tumor Cells is Associated With a Favorable Prognosis in Patients With High-Risk Endometrial Cancer. Gynecol Oncol (2021) 162(3):631–7. doi: 10.1016/j.ygyno.2021.07.009 34272092

[33] Yamashita H Nakayama K Ishikawa M Ishibashi T Nakamura K Sawada K. Relationship Between Microsatellite Instability, Immune Cells Infiltration, and Expression of Immune Checkpoint Molecules in Ovarian Carcinoma: Immunotherapeutic Strategies for the Future. Int J Mol Sci (2019) 20(20). doi: 10.3390/ijms20205129 PMC6829575 31623180

[34] Özcan D Lade-Keller J Tramm T. Can Evaluation of Mismatch Repair Defect and TILs Increase the Number of Triple-Negative Breast Cancer Patients Eligible for Immunotherapy? Pathol Res Pract (2021) 226:153606. doi: 10.1016/j.prp.2021.153606 34530255

[35] Zurlo IV Schino M Strippoli A Calegari MA Cocomazzi A Cassano A. Predictive Value of NLR, TILs (CD4+/CD8+) and PD-L1 Expression for Prognosis and Response to Preoperative Chemotherapy in Gastric Cancer. Cancer Immunol Immunother: CII (2022) 71(1):45–55. doi: 10.1007/s00262-021-02960-1 34009410 PMC8738448

[36] Hongo T Yamamoto H Jiromaru R Yasumatsu R Kuga R Nozaki Y. PD-L1 Expression, Tumor-Infiltrating Lymphocytes, Mismatch Repair Deficiency, EGFR Alteration and HPV Infection in Sinonasal Squamous Cell Carcinoma. Modern Pathol (2021) 34(11):1966–78. doi: 10.1038/s41379-021-00868-w 34218257

[37] Guo W Zhang F Shao F Wang P Li Z Yang X. PD-L1 Expression on Tumor Cells Associated With Favorable Prognosis in Surgically Resected Esophageal Squamous Cell Carcinoma. Hum Pathol (2019) 84:291–8. doi: 10.1016/j.humpath.2018.09.014 30296523

[38] Horimoto Y Thinzar Hlaing M Saeki H Kitano S Nakai K Sasaki R. Microsatellite Instability and Mismatch Repair Protein Expressions in Lymphocyte-Predominant Breast Cancer. Cancer Sci (2020) 111(7):2647–54. doi: 10.1111/cas.14500 PMC7385389 32449246

[39] Dong D Lei H Liu D Bai H Yang Y Tang B. POLE and Mismatch Repair Status, Checkpoint Proteins and Tumor-Infiltrating Lymphocytes in Combination, and Tumor Differentiation: Identify Endometrial Cancers for Immunotherapy. Front Oncol (2021) 11:640018. doi: 10.3389/fonc.2021.640018 33816285 PMC8017289

[40] Song Y Gu Y Hu X Wang M He Q Li Y. Endometrial Tumors With MSI-H and dMMR Share a Similar Tumor Immune Microenvironment. OncoTargets Ther (2021) 14:4485–97. doi: 10.2147/OTT.S324641 PMC8379685 34429613

[41] Bie F Tian H Sun N Zang R Zhang M Song P. Comprehensive Analysis of PD-L1 Expression, Tumor-Infiltrating Lymphocytes, and Tumor Microenvironment in LUAD: Differences Between Asians and Caucasians. Clin Epigenetics (2021) 13(1):229. doi: 10.1186/s13148-021-01221-3 34933667 PMC8693498

[42] Shirasawa M Yoshida T Shimoda Y Takayanagi D Shiraishi K Kubo T. Differential Immune-Related Microenvironment Determines Programmed Cell Death Protein-1/Programmed Death-Ligand 1 Blockade Efficacy in Patients With Advanced NSCLC. J Thorac Oncol (2021) 16(12):2078–90. doi: 10.1016/j.jtho.2021.07.027 34419685

