# Supplementary figures and images for: PMS2 Expression With Combination of PD-L1 and TILs for Predicting Survival of Esophageal Squamous Cell Carcinoma
Source: Front Oncol. 2022 Jul 5;12:897527. doi: 10.3389/fonc.2022.897527 (PMC9294642; doi:10.3389/fonc.2022.897527)

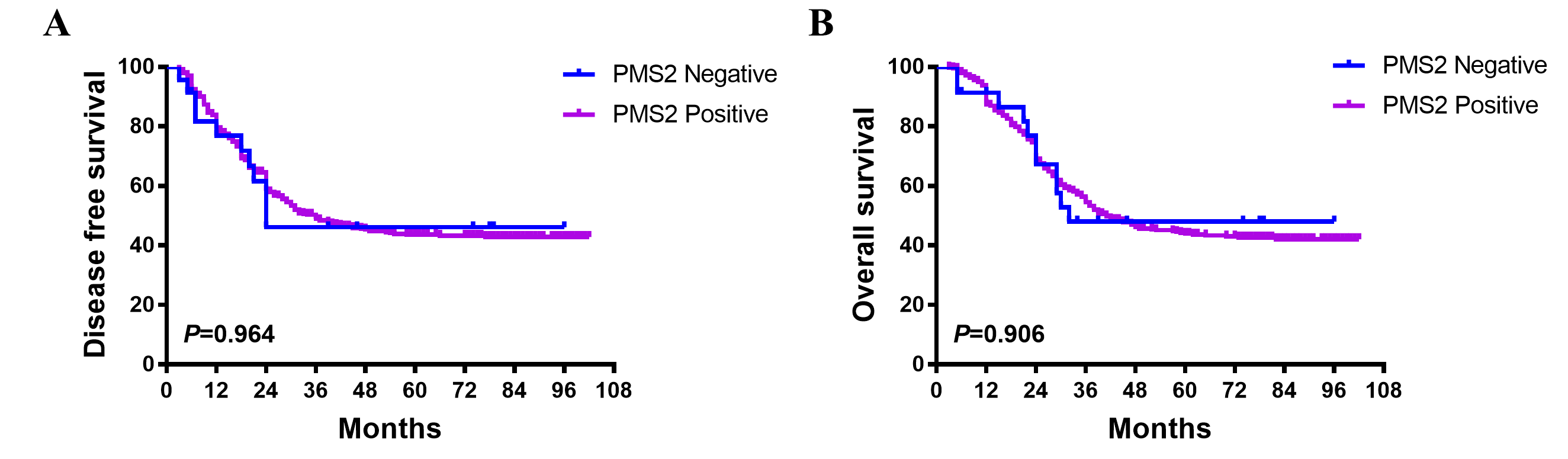

Supplement: Supplementary Figure 1 — (A, B) There was no association between PMS2 deficiency and DFS (P=0.964) or OS (P=0.906). [file Image_1.tif]

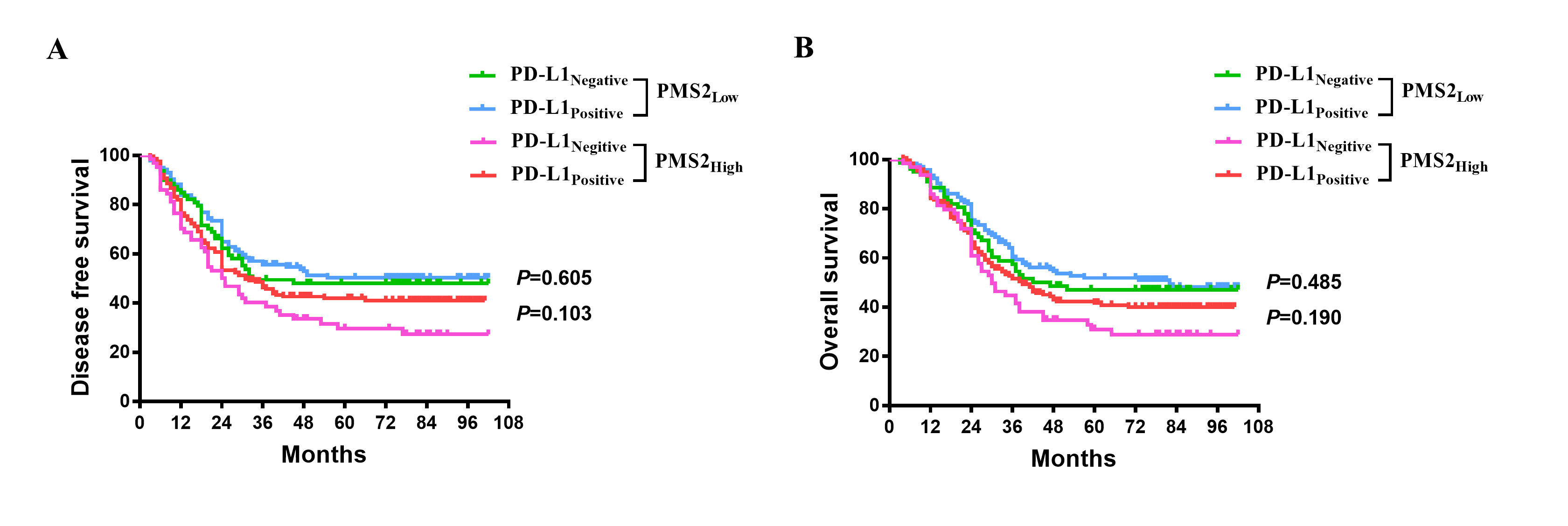

Supplement: Supplementary Figure 2 — (A, B) In subgroup analyses for patients with high PMS2 expression, patients with PD-L1 expression tended to have better DFS (P=0.103) and OS (P=0.190) than those without PD-L1 expression, which were not found in subgroup analyses for patients with low PMS2 expression. [file Image_2.tif]
